# Novel parent-of-origin-specific differentially methylated loci on chromosome 16

**DOI:** 10.1186/s13148-019-0655-8

**Published:** 2019-04-08

**Authors:** Katharina V. Schulze, Przemyslaw Szafranski, Harry Lesmana, Robert J. Hopkin, Aaron Hamvas, Jennifer A. Wambach, Marwan Shinawi, Gladys Zapata, Claudia M. B. Carvalho, Qian Liu, Justyna A. Karolak, James R. Lupski, Neil A. Hanchard, Paweł Stankiewicz

**Affiliations:** 1 Department of Molecular and Human Genetics, Baylor College of Medicine, Houston, TX USA; 2 Division of Human Genetics, Cincinnati Children’s Hospital Medical Center, University of Cincinnati College of Medicine, Cincinnati, OH USA; 3 Pediatrics, Northwestern University Feinberg School of Medicine, Chicago, IL USA; 4 Division of Newborn Medicine, Edward Mallinckrodt Department of Pediatrics, Washington University School of Medicine, St. Louis, MO USA; 5 Division of Genetics and Genomic Medicine, Edward Mallinckrodt Department of Pediatrics, Washington University School of Medicine, St. Louis, MO USA; 6 Department of Pediatrics, Baylor College of Medicine, Houston, TX USA; 7 Texas Children’s Hospital, Houston, TX USA; 8 USDA/ARS/Children’s Nutrition Research Center, Baylor College of Medicine, Houston, TX USA

**Keywords:** Imprinting, CpG, Trisomy 16, Uniparental disomy 16, ACDMPV

## Abstract

**Background:**

Congenital malformations associated with maternal uniparental disomy of chromosome 16, upd(16)mat, resemble those observed in newborns with the lethal developmental lung disease, alveolar capillary dysplasia with misalignment of pulmonary veins (ACDMPV). Interestingly, ACDMPV-causative deletions, involving *FOXF1* or its lung-specific upstream enhancer at 16q24.1, arise almost exclusively on the maternally inherited chromosome 16. Given the phenotypic similarities between upd(16)mat and ACDMPV, together with parental allelic bias in ACDMPV, we hypothesized that there may be unknown imprinted loci mapping to chromosome 16 that become functionally unmasked by chromosomal structural variants.

**Results:**

To identify parent-of-origin biased DNA methylation, we performed high-resolution bisulfite sequencing of chromosome 16 on peripheral blood and cultured skin fibroblasts from individuals with maternal or paternal upd(16) as well as lung tissue from patients with ACDMPV-causative 16q24.1 deletions and a normal control. We identified 22 differentially methylated regions (DMRs) with ≥ 5 consecutive CpG methylation sites and varying tissue-specificity, including the known DMRs associated with the established imprinted gene *ZNF597* and DMRs supporting maternal methylation of *PRR25*, thought to be paternally expressed in lymphoblastoid cells. Lastly, we found evidence of paternal methylation on 16q24.1 near *LINC01082* mapping to the *FOXF1* enhancer.

**Conclusions:**

Using high-resolution bisulfite sequencing to evaluate DNA methylation across chromosome 16, we found evidence for novel candidate imprinted loci on chromosome 16 that would not be evident in array-based assays and could contribute to the birth defects observed in patients with upd(16)mat or in ACDMPV.

**Electronic supplementary material:**

The online version of this article (10.1186/s13148-019-0655-8) contains supplementary material, which is available to authorized users.

## Introduction

Trisomy 16, the most frequent prenatally detected trisomy [1], is embryonic lethal unless rescued during early embryogenesis. Maternal uniparental disomy of chromosome 16, upd(16)mat, in humans is among the most commonly identified chromosomal UPDs [2, 3], typically arising during embryonic development after the paternal chromosome is lost, which occurs in a third of all trisomy 16 rescue events. upd(16)mat has been associated with intrauterine growth restriction (IUGR) and a variety of congenital malformations, including heart defects, pulmonary hypoplasia, tracheoesophageal fistula, gut malrotation, absent gall bladder, renal agenesis, hydronephrosis, imperforate anus, and single umbilical artery [2]. Of these clinical features, IUGR has been most often attributed to confined placental trisomy 16, although growth restrictions and short stature have been reported in association with other chromosomal UPDs, including the first human case [4], whereas the other phenotypic features have been suggested to result from upd(16) or embryonic mosaic trisomy 16 [5–9].

Trisomy 16 is most often the result of maternal meiosis I nondisjunction; consequently, most upd(16)mat cases are heterodisomic, retaining both distinct maternal copies of chromosome 16 after trisomic rescue. Whereas genetic recombination between the homologous chromosomes can result in regions of homozygosity with the potential to unmask recessive disorders [10], the vast majority of homologous loci mapping to chromosome 16 in upd(16)mat are non-identical [11]. Consequently, the clinical phenotypes associated with upd(16)mat are likely the result of unbalanced expression of imprinted genes. In contrast, the very rare paternal uniparental disomy of chromosome 16, upd(16)pat, has been associated with a relatively benign clinical phenotype attributed to duplication of the paternal chromosome as a means of rescuing maternal nullisomy 16. Since most upd(16)pat cases are isodisomic, gene-dosage dependent congenital malformations found in upd(16)pat are typically attributed to unmasked mutations in recessive disease genes [12].

Genome-wide searches for imprinted regions in different tissues have been performed through DNA sequence-based computational predictions [13], gene expression assays [14–18], DNA methylation analyses [19–23], and combinations thereof [24]. The imprinted locus, most consistently identified as such on chromosome 16, is the paternally imprinted (maternally expressed) *ZNF597* gene, which encodes a zinc finger protein. While the biological function of *ZNF597* is unknown, Inoue et al. [25] have suggested that its overexpression might contribute to some of the clinical phenotypes associated with upd(16)mat.

Interestingly, with the exception of IUGR, many of the clinical features seen in patients with upd(16)mat have often been observed also in children with the neonatal lethal lung developmental disorder, alveolar capillary dysplasia with misalignment of pulmonary veins (ACDMPV, MIM #265380) [26]. Aside from pulmonary hypoplasia, the malformations observed both in infants with ACDMPV and upd(16)mat affect the respiratory system (e.g., tracheoesophageal fistula, tracheal narrowing), gastrointestinal system (e.g., intestinal malrotation, omphalocele, gastrointestinal atresias, imperforate anus), the cardiovascular system (e.g., ventricular and atrial septal defects, coarctation of the aorta), and the genitourinary system (e.g., hydronephrosis, hydroureter, hypospadias) [7, 27–29]. ACDMPV is caused by mutations or copy-number variant (CNV) deletions involving the *FOXF1* locus at 16q24.1 [29, 30]. Interestingly, deletions of the *FOXF1* upstream enhancer have been found almost exclusively on the maternally inherited chromosome 16 [31, 32]. Given the parental allelic bias in ACDMPV as well as the phenotypic differences between maternal and paternal upd(16), we hypothesized that there may be unknown imprinted or partially imprinted loci mapping to chromosome 16q24.1 that contribute to the observed key clinical phenotypic features of both upd(16)mat and ACDMPV.

To investigate this hypothesis and to identify novel differentially methylated regions (DMRs), we assessed DNA methylation at CpG dinucleotides across chromosome 16 using capture-based targeted bisulfite sequencing in samples with maternal and paternal upd(16) and large heterozygous deletions at 16q23.3q24.1.

## Methods

### Subjects

Peripheral blood samples were obtained from two individuals with upd(16)mat, two with upd(16)pat, and two normal controls. Both upd(16)mat blood samples were largely heterodisomic in the pericentromeric regions with distal isodisomies on both chromosome arms, while both upd(16)pat samples were suspected to be fully isodisomic (Additional file 1: Table S1). There was no evidence for mosaic trisomy 16 using chromosomal microarray, karyotyping, or exome analyses in one upd(16)mat proband. The placenta for the same upd(16)mat proband was noted to be small, but no cytogenetic analyses were performed to test for trisomy 16. Information regarding trisomy 16 testing was not available for the remaining upd(16) cases. DNA was extracted from blood using Gentra Puregene Kit (Qiagen, Germantown, CA, USA).

Skin fibroblasts were collected from two additional individuals with upd(16)mat and two additional normal controls. The upd(16)mat sample that passed subsequent quality measures was heterodisomic in the pericentromeric regions with one or two distal isodisomies (Additional file 1: Table S1). No evidence of trisomy 16 was detected in this proband using fluorescence in situ hybridization (FISH); however, FISH analysis of placental tissue indicated confined placental trisomy 16 mosaicism. Fibroblasts were cultured in Dulbecco’s Modified Eagle Medium (DMEM) supplemented with 10% fetal bovine serum (FBS). Fibroblast DNA was extracted using the Gentra Puregene Kit (Qiagen).

Autopsy lung tissue samples were obtained from two ACDMPV individuals with heterozygous CNV deletions (86,505,450-86,575,461 and 83,673,382-86,298,284, GRCh37/hg19) on the maternally inherited chromosome 16 and one age-matched control individual with a medical condition unrelated to lung development. DNA was extracted from frozen lung tissues using DNeasy Blood and Tissue Kit (Qiagen). Detailed patient information was previously published [12, 29, 32, 33] or can be found in the Additional file 2.

### Bisulfite sequencing

Bisulfite sequencing of chromosome 16 was performed according to manufacturer’s instructions (protocol v1.1) using a custom SeqCap Epi Enrichment Kit (Roche NimbleGen, Madison, WI, USA) designed to capture 74.1–85.6% of CpG sites on chromosome 16. Briefly, 1 μg of genomic DNA was fragmented using a Covaris Ultra Sonicator (Covaris Inc., Woburn, MA, USA) to a size range of 180–220 bp. Following DNA fragmentation, sample libraries were prepared using the KAPA Library Preparation Kit for Illumina Platforms (Kapa Biosystems, Boston, MA, USA), according to manufacturer’s protocol v2.14. DNA was subjected to end repair, A-tailing, and adapter ligation on SeqCap EZ Purification Beads (Roche NimbleGen). Post-ligation cleanup was performed using AMPure XP Beads (Agencourt, Beverly, MA, USA). DNA sample libraries were bisulfite converted using EZ DNA Methylation-Lightning Kit (Zymo Research, Irvine, CA, USA) at 98 °C for 8 min, followed by 60 min incubation at 54 °C, and purification on Zymo Spin IC columns. Bisulfite-converted sample libraries were amplified using pre-capture ligation-mediated polymerase chain reaction (LM-PCR) and purified using AMPure XP Beads (Agencourt). The quality of the amplified bisulfite-converted sample libraries was checked on Bioanalyzer 2100 using DNA 1000 chip (Agilent Technologies, Santa Clara, CA, USA). One microgram of the amplified bisulfite-converted sample libraries was then hybridized to SeqCap Epi Probe Pool (Roche NimbleGen) at 47 °C for 64 h. Captured bisulfite-converted samples were washed and recovered using SeqCap Pure Capture Beads (Roche NimbleGen) and subsequently amplified using LM-PCR. Purification of the amplified and captured bisulfite-converted DNA samples was performed using AMPure XP Beads (Agencourt) followed by quantification and quality assessment using a Bioanalyzer DNA 1000 chip (Agilent Technologies). The captured and amplified DNA fragments were then sequenced using 100 bp paired-end reads on the Illumina HiSeq 2000 platform (Illumina Inc., San Diego, CA, USA), multiplexed to up to six samples per lane.

### Data preprocessing

Samples were demultiplexed using Casava (v1.8, Illumina). Thereafter, Bismark (v0.12.3) [34] was used to (1) align FASTQ files to the hg19 reference genome, (2) remove duplicate reads, (3) trim five bases off the 5′ ends of all reads and one base off the 3′ end of “read 2” paired reads in order to eliminate known methylation biases, and (4) extract counts of methylated and unmethylated cytosines found on the forward and reverse strand in a CpG dinucleotide context.

### Peripheral blood-based data analysis

Methylated and unmethylated cytosine counts for each sample were loaded into the R software environment (v3.4.4). To increase coverage, counts at the same locus were pooled for all peripheral blood samples of the same disomy category, upd(16)mat, upd(16)pat, and biparental control, and only those loci represented in all categories were used for subsequent analyses. An absolute difference in percent methylation of ≥ 40 between maternal and paternal upd(16) and an intermediate methylation in control (biparental) samples (upd(16)mat > biparental > upd(16)pat or upd(16)mat < biparental < upd(16)pat) at individual loci was considered evidence of parent-of-origin biases in methylation. Adjacent differentially methylated loci with the same direction of effect were considered as regions; neighboring regions that were separated by only one discordant methylation site were combined into one region. Regions with a median coverage < 10× in any sample category were removed, as were those with an absolute difference in percent methylation > 50 between samples of the same category, if they had a median coverage > 3×. Results from peripheral blood samples were then compared to DNA methylation of fibroblast cells and three lung tissue samples.

### Lung tissue-based data analysis

Since maternal and paternal upd(16) lung tissue samples were not available, ACDMPV samples with deletions of the maternal 16q23.3q24.1 allele were used instead to study parent-of-origin biases in methylation based on the remaining, hemizygous, paternal alleles. Percent methylation was compared at CpG methylation sites that were captured at least once per sample across each deleted locus. For this analysis, an absolute difference in percent methylation ≥ 20 between the hemizygous ACDMPV sample and each dizygotic control was considered suggestive of parent-of-origin biases in methylation. Adjacent CpG methylation sites that matched this criterion and had the same direction of effect were collapsed into regions; regions containing at least five differentially methylated cytosines and a median coverage of 7× were considered candidate loci for parent-of-origin biased DNA methylation. In comparison to the 10× coverage threshold from the blood-based analysis, this more lenient coverage filter was chosen to account for the fact that samples were not collapsed by case/control-status in the lung-based analysis.

### Variant calling

Single nucleotide variants (SNVs) were called from aligned, coordinate-sorted, and deduplicated BAM files using Bis-SNP (v1.0.0) [35]. The software’s BisulfiteGenotyper was supplied with the hg19 reference genome and a variant file of common single nucleotide polymorphisms (dbSNP build 151). Raw SNV calls were then filtered using Bis-SNP’s VCFpostprocess, with default parameters, to produce a final list of SNVs for each sample. To identify regions of homozygosity for the purpose of gauging hetero- and isodisomy, the resulting variant call files (VCFs) were run through the bcftools *roh* algorithm (v1.8) with the parameters -G30 and --AF-dflt 0.4. The only samples to include at least one region of homozygosity larger than 10 Mb, indicative of isodisomy [36], were both upd(16)pat peripheral blood samples and one upd(16)mat fibroblast sample that passed filtering thresholds.

## Results

### Bisulfite sequencing in upd(16) samples confirms and expands differential methylation of CpGs at known imprinted regions

We performed chromosome 16-wide targeted capture-based bisulfite sequencing on DNA samples extracted from the peripheral blood of two individuals with upd(16)mat, two with upd(16)pat, and two normal controls. In addition, we used the same bisulfite sequencing approach on skin fibroblast DNA obtained from two individuals with upd(16)mat and two healthy controls; however, only one fibroblast sample, upd(16)mat, passed our quality control filters. Consequently, in our subsequent analyses, we primarily compared DNA methylation between different UPD states in peripheral blood samples and then juxtaposed the identified methylation patterns alongside those in the fibroblast upd(16)mat sample. By collapsing the sequencing reads of peripheral blood samples within each category to strengthen depth of coverage, we were able to capture 1,690,963 forward and reverse strand CpG cytosines that were covered in all sample groups. In contrast to the widely used Illumina Infinium 450 K and EPIC methylation arrays, which, respectively, target 2.0% and 3.4% of CpGs on chromosome 16, our approach allowed us to assess DNA methylation at a much higher resolution by capturing 76.7% of CpG sites (Additional file 2**:** Fig. S1).

**Fig. 1:**
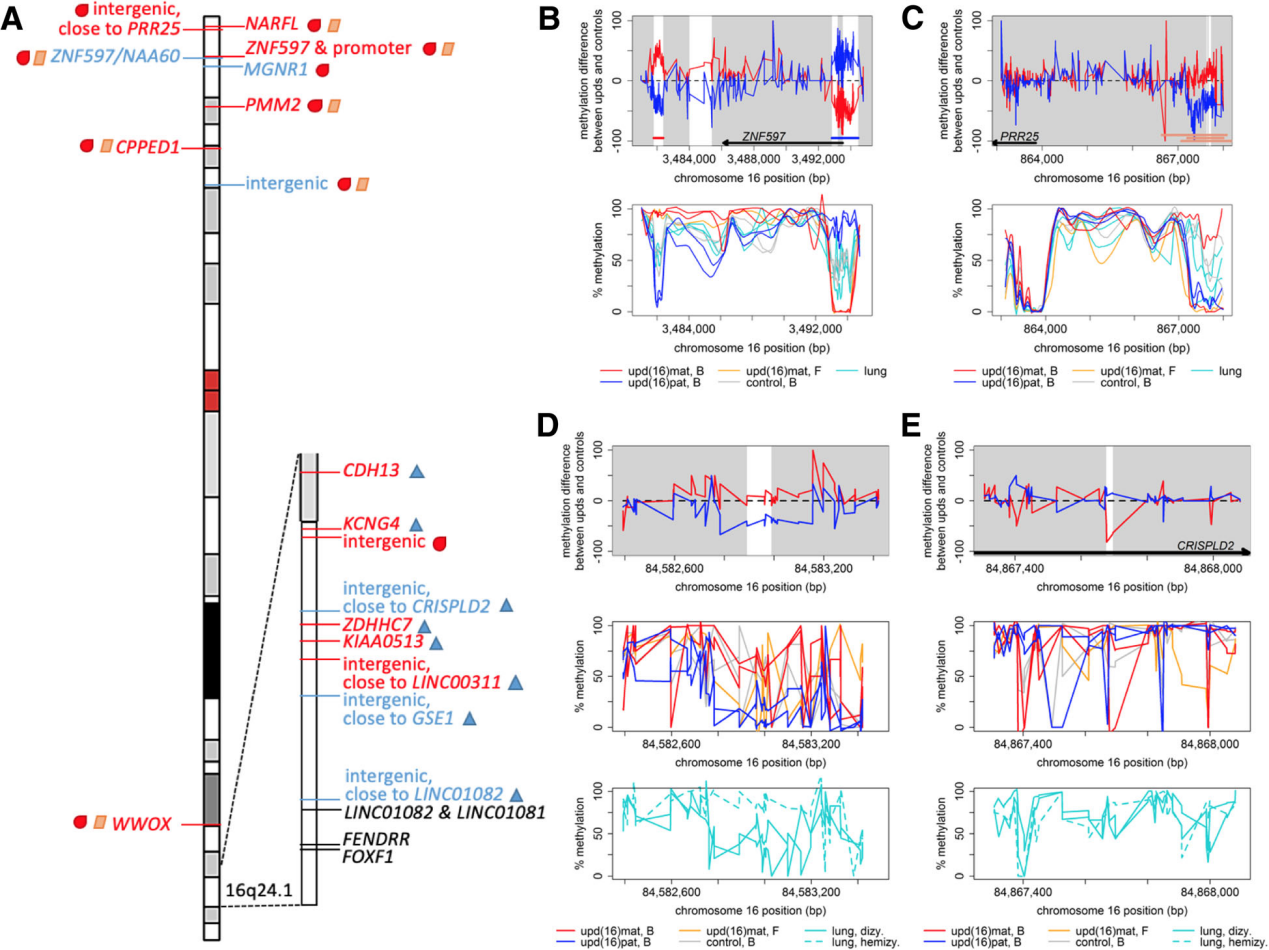
Parent-of-origin biased differential DNA methylation on chromosome 16. **a** Regions with preferential maternal methylation are written in red font, those with paternal methylation in blue. Symbols next to gene names associated with differentially methylated regions indicate the tissue in which the parent-of-origin biases in methylation could be observed (teardrop, blood; triangle, lung; diamond, fibroblast). **b** Differential methylation at known imprinted locus *ZNF597*. Horizontal bars indicate known regions of differential methylation (red, maternal methylation; blue, paternal methylation). **c** Differential methylation at suspected imprinted locus near *PRR25* (pink horizontal bars). Differential methylation at 16q24.1 *TLDC1*/*COTL1*-intergenic locus (**d**) and *CRISPLD2* (**e**). White areas in **b–e** indicated regions suggestive of parent-of-origin biased methylation based on our analysis, gray-shaded areas fall outside these boundaries. Lines in **b–e** bottom panels were created using LOESS smoothing with a span of 0.1, causing some data points to extend beyond the expected 0–100% methylation range. B blood, F fibroblast, L lung

We then searched for parent-of-origin biases in methylation, which we defined as an absolute difference in percent methylation of at least 40 between upd(16)mat and upd(16)pat with intermediate methylation in control samples. Among the 25,105 CpG methylation sites matching these criteria, we found 14 genomic regions with a median read coverage ≥ 10× and at least five adjacent differentially methylated cytosines with evidence of parent-of-origin biased methylation (Table 1, Fig. 1a, Additional file 1**:** Table S2, Additional file 2**:** Fig. S2). The three regions containing most of the differentially methylated cytosines coincided with the known chromosome 16 imprinted gene *ZNF597* (Fig. 1b)—two with the paternally methylated region intergenic to *ZNF597* and *NAA60* on 16p13.3, methylation at which serves as a bidirectional repressor [20], and the other with the maternally methylated region telomeric to *ZNF597* [21]. Lastly, we identified an additional, smaller maternally methylated region in the last exon of *ZNF597*, which has not been previously described. This region might have escaped prior detection due to the absence of overlapping Infinium methylation array probes or the relatively low CpG density. Methylation at these imprinted regions showed the same parent-of-origin biased pattern in the upd(16)mat fibroblast sample, suggesting that the methylation patterns at these imprinted regions are conserved across tissues.

**Table 1:**
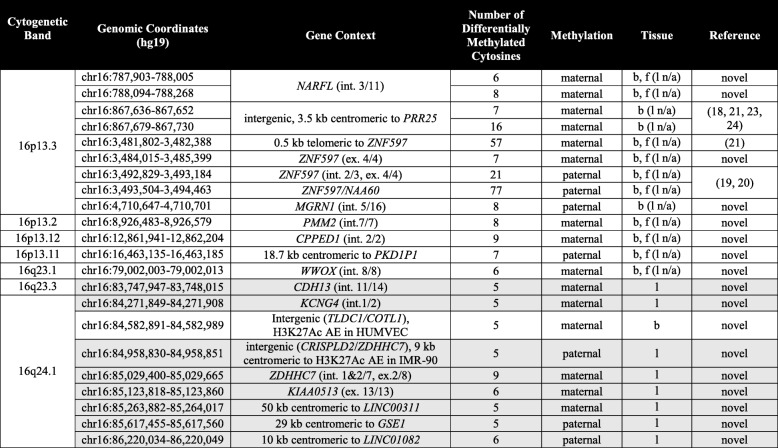
Genomic regions with evidence of parent-of-origin biased methylation on chromosome 16

Not shaded entries were found with blood-based analysis, gray-shaded entries with lung-based analysis. *Abbreviations*: *int* intron, *ex* exon, *nc* non-coding, *AE* histone modification indicative of the active enhancer, *b* peripheral blood, *f* skin fibroblasts, *l* lung, *n*/*a* data not available

### Parent-of-origin biased methylation provides evidence for imprinting of *PRR25* in peripheral blood

Among the regions with suggestive evidence of parent-of-origin biased methylation were two maternally methylated intergenic regions between *PRR25* and *LMF1* at 16p13.3 that were within 27 bp of each other (Table 1, Fig. 1c). Although this locus has not been reported as imprinted as consistently as *ZNF597*, data from previous studies show maternal methylation at chr16:867,208–868,000 [21], chr16:866,647–868,075 [23], and chr16:867,075–868,443 [24] overlapping the *PRR25*/*LMF1* intergenic region identified in our study. In addition, Jadhav et al. [18] presented evidence that *PRR25* is paternally expressed in lymphoblastoid cells, suggesting that methylation of the maternal allele at this locus could act as a suppressor of *PRR25* expression in *cis*. We were unable to find any published information on *LMF1* imprinting, potentially indicating that the methylation at this locus, intergenic to *PRR25* and *LMF1*, might only affect *PRR25*. Furthermore, the methylation patterns in upd(16)mat fibroblast samples did not resemble those in maternal UPD blood samples, implying that the methylation at this *PRR25* locus might be tissue-specific.

### Blood-based analysis reveals two small, tissue-specific loci with evidence of parent-of-origin biased DNA methylation on 16q24.1

In addition, we identified evidence for a small maternally methylated region on 16q24.1 in blood cells (Table 1, Fig. 1d). This differentially methylated intergenic region is flanked by *TLDC1* (TBC/LysM-associated domain-containing protein 1) and *COTL1* (coactosin like F-actin binding protein 1), neither of which have been associated with ACDMPV, regulation of *FOXF1* expression, or causatively linked to any other Mendelian disorders. While DNA methylation levels were higher in upd(16)mat relative to upd(16)pat and controls in peripheral blood samples, DNA methylation levels were lower in the upd(16)mat fibroblast sample, suggesting that parent-of-origin biased DNA methylation at this intergenic 16q24.1 locus might be tissue-specific.

We then sought to evaluate the methylation status of this locus in lung tissues. We performed bisulfite sequencing on three lung tissue samples, one control and two obtained from individuals with ACDMPV caused by heterozygous, different-sized 16q24.1 deletions of the maternally inherited chromosome 16. Consequent upon the maternal deletion, one of the ACDMPV lung samples was hemizygous for the paternal chromosome at the 16q24.1 *TLDC1*/*COTL1*-intergenic locus. We found that at this locus, paternal DNA methylation levels in lung tissue were high with intermediate methylation in biparental controls (Fig. 1d). These results are in contrast to those seen in blood, indicating that the initially identified methylation patterns at this *TLDC1*/*COTL1* intergenic locus might be blood-specific. The differences in methylation in lung tissue compared to blood, i.e., paternal > biparental methylation versus paternal < biparental < maternal methylation, might further indicate an inversion of the parentally biased methylation patterns between these tissues.

Given our particular interest in DMRs at 16q24.1, we next took a closer look at regions on this chromosome band with at least two, but fewer than five, neighboring differentially methylated CpG cytosines in peripheral blood samples that would have been below filtering thresholds in our previous analyses. Since SNVs occur frequently at CpG dinucleotides and can obscure methylation calls [37, 38], we eliminated eight of these smaller regions that were found to have SNVs at candidate CpGs based on the variant calling results from our bisulfite sequencing data. We focused on those regions that (1) overlapped a hemizygous paternal allele in ACDMPV lung samples, (2) showed resembling parent-of-origin biased methylation in lung tissue, and (3) spanned more than one CpG dinucleotide. Using this analytical approach, we identified one additional 16q24.1 region—chr16:84,867,676–84,867,696—with evidence of parent-of-origin biased DNA methylation in both blood and lung tissue. This locus showed evidence of paternal DNA methylation at two neighboring CpG dinucleotides located in the first intron of *CRISPLD2* (Fig. 1e), which encodes a cysteine-rich secretory protein and has been associated with non-syndromic cleft lip [39]. Upd(16)mat fibroblast DNA methylation levels were high, in contrast to upd(16)mat blood samples (Fig. 1e), indicating that the parent-of-origin methylation at this locus might be specific to blood and lung tissue.

### Lung-based analysis of 16q24.1 reveals evidence of parent-of-origin biased DNA methylation

**Fig. 2:**
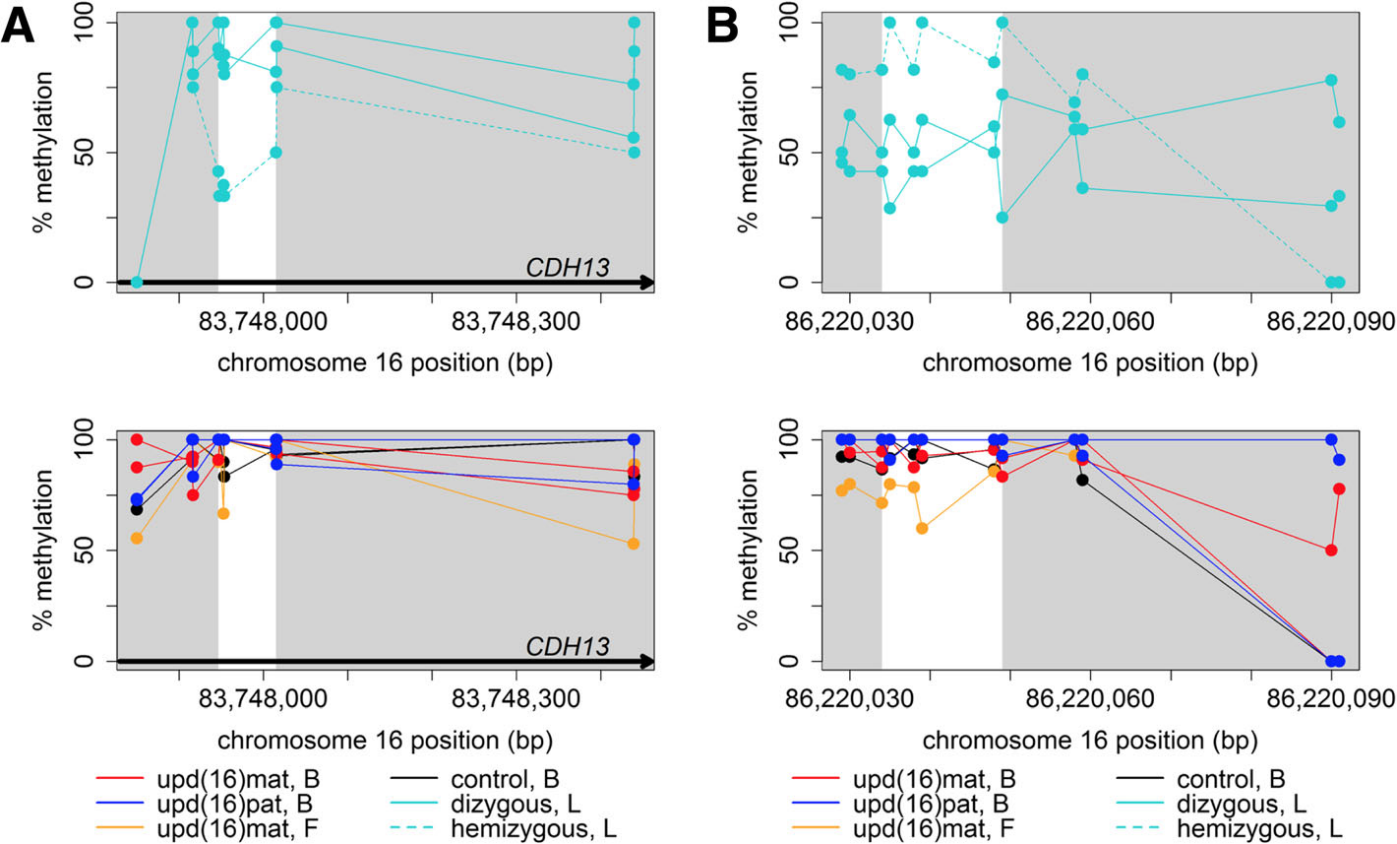
Methylation patterns at new loci with evidence of parent-of-origin biased methylation in lung tissue. **a** DNA methylation at a region overlapping *CDH13*. **b** DNA methylation at a region within 10 kb of *LINC01082*. White areas indicate regions suggestive of parent-of-origin biased methylation based on our analysis, gray-shaded areas fall outside these boundaries. Top panels show DNA methylation in lung tissue samples; bottom panels show DNA methylation in blood and fibroblast samples for comparison

To find regions with parent-of-origin biased methylation specific to lung tissue, which would not have been identified in the previous peripheral blood-based analyses, we next studied DNA methylation at the maternally deleted locus, which exposes the remaining paternal allele, in each of the two ACDMPV lung samples. Since the deleted segments in each sample do not overlap, we compared CpG methylation of the hemizygous segment from each ACDMPV sample with those of the equivalent dizygous segments from the remaining ACDMPV and lung control samples. CpG methylation loci with a difference in percent methylation of at least 20 between our case and each dizygous control were considered indicative of parent-of-origin biased DNA methylation. Interestingly, we did not identify any patterns matching our criteria for parent-of-origin biased DNA methylation within chr16:86,505,450–86,575,461, the smaller of the two maternally deleted regions containing *FOXF1*, its neighboring long noncoding RNA (lncRNA) *FENDRR*, or their promoters. However, the larger deleted locus at chr16:83,673,382–86,298,284, containing the *FOXF1* enhancer, harbored eight intervals with evidence of parent-of-origin biased methylation (Table 1, Additional file 1**:** Table S3, Additional file 2**:** Fig. S3). One of these intervals overlapped an intron of the H-cadherin-encoding gene *CDH13* (Fig. 2a), for which aberrant promoter methylation and consequent loss of gene expression have been associated with non-small cell lung carcinoma [40]. Further, we identified a small intergenic region within 10 kb of the transcription start site of *LINC01082* (Fig. 2b)—a lncRNA which is thought to function as a transcriptional regulator of *FOXF1* and the loss of which has been associated with atypical ACDMPV [41].

## Discussion

We studied the genomic methylation pattern of chromosome 16 at high-resolution to identify new loci with parent-of-origin biased DNA methylation that might explain some of the phenotypic similarities between upd(16)mat and ACDMPV as well as differences between upd(16)mat and upd(16)pat.

As a positive control and experimental validation of our analytical approach, we confirmed known DMRs associated with *ZNF597*, the best-documented imprinted gene on chromosome 16: the maternally methylated locus mapping 0.5 kb telomeric to the *ZNF597* gene and the paternally methylated locus between *ZNF597* and *NAA60*, both on 16p13.3 [19–21]. We identified an additional maternally methylated interval in the *ZNF597*/*NAA60* intergenic region, which is likely an extension of the already known locus. Moreover, we have found further evidence in support of blood-specific maternal methylation at *PRR25* on 16p13.3 [18, 21, 23, 24]. However, the function and therefore potential pathogenic relevance of *PRR25* remains unknown.

A focused DNA methylation analysis of the hemizygous 16q24.1 region in lung tissue samples from individuals with ACDMPV caused by deletions of the maternal allele of this region revealed a putatively paternally methylated locus near *LINC01082*. *LINC01082* is located within the *FOXF1* lung-specific upstream enhancer and has been proposed to regulate *FOXF1* expression [32, 41]. We hypothesize that parent-of-origin biased methylation of this site may contribute to differential expression of *LINC01082* and thus differential expression of *FOXF1* as well. Functionality of this differentially methylated locus is additionally supported by the presence of conserved binding sites for several transcription factors, including lung-specific LUN1 [29].

Recently, based on qRT-PCR and bisulfite-PCR, Alsina Casanova et al. [42] concluded that there was no evidence of imprinting at the 16q24.1 locus in lung tissue. However, due to a lack of informative SNVs, allelic expression of *LINC01082* was not assessed. Further, their differential methylation analyses relied upon mean methylation levels of 20 CpGs, a window size with which differential methylation of regions containing fewer CpGs, such as the one near *LINC01082*, would not have been detected. Nevertheless, they showed regions consistent with allelic methylation in lung overlapping *LINC01082* and noted sperm-specific methylation across a ~ 250 kb region, including *FOXF1* and its intronic enhancer [43], which appears to become mostly unmethylated in blastocysts. The evidence of parent-of-origin biased DNA methylation identified in the vicinity of *LINC01082* could therefore be a remnant of gamete-specific methylation that persisted after preimplantation reprogramming, as is typically observed for germline derived DMRs. Alternatively, it could reflect confinement of differentially methylated regions to a small fraction of specialized lung cells.

In the analysis of ACDMPV lung samples, we also identified evidence for a paternally methylated intergenic interval between *CRISPLD2* and *ZDHHC7* on 16q24.1. Of note, during a targeted analysis of the 16q24.1 region in peripheral blood, we found a small locus in the first intron of *CRISPLD2* with evidence of paternal methylation that was also reflected in lung tissue. *CRISPLD2* has been reported in the context of non-syndromic cleft palate. While neither upd(16)mat nor ACDMPV individuals present with cleft palate, it would be interesting to determine whether individuals with ACDMPV caused by larger-sized deletions that span *CRISPLD2* share specific clinical phenotypes and how these phenotypes compare to those commonly observed in individuals with upd(16)mat.

Approximately 2 Mb centromeric to *FOXF1*, we found a maternally methylated intergenic interval between *TLDC1* and *COTL1*. Sanchez-Delgado et al. [23] described two regions associated with *TLDC1* that showed gamete-specific methylation patterns—one at the *TLDC1* promoter and the other intergenic to *TLDC1* and *COTL1,* mapping approximately 13 kb upstream to our candidate interval. Both regions are methylated in oocytes and unmethylated in sperm; however, differentiated tissues appear to become fully methylated at the intergenic locus, while intermediate methylation levels are maintained at the *TLDC1* promoter locus. The maternally methylated *TLDC1*/*COTL1* intergenic interval identified in our study could be a remnant of the transient gamete-specific methylation observed in the genomic region surrounding *TLDC1*. Even though parent-of-origin methylation in this region was specific to blood, other tissues not sampled in the context of this study, but relevant to the shared upd(16)mat and ACDMPV phenotypes, could show a similar differential methylation signature.

While we identified suggestive evidence of parent-of-origin biases in DNA methylation at 16q24.1, it is also possible that larger imprinted loci exist in this region whose differential methylation might have been lost later during embryonic development. Sanchez-Delgado et al. [23] listed 17 regions on 16q24.1 that are methylated in oocytes but not in sperm and showed intermediate methylation levels in the blastocyst stage and often in the placenta. The differentially methylated region closest to *FOXF1* was an intergenic locus at chr16:86,638,847–86,639,716. If maternal methylation at these regions acts as a repressor of gene expression and the tissues of early development rely mostly on the expression of the paternal allele, then pathogenic deletions of the maternal allele causing ACDMPV would be of smaller consequence with the potential to result in a live birth, whereas deletions of the paternal allele would potentially have more severe phenotypic/developmental consequences. Targeted analyses of 16q24.1 in germ cells and placental tissue might provide more insight into a possibly transient nature of the imprinting surrounding *FOXF1*.

Aside from the 16q24.1 loci described above, we found additional regions on chromosome 16 in peripheral blood with evidence for parent-of-origin biased methylation that might be of relevance to the upd(16)mat phenotype, including a maternally methylated region within the last intron of *PMM2* on 16p13.2. *PMM2* encodes phosphomannomutase-2 that has been associated with congenital disorder of glycosylation type 1a (MIM #212065). Among others, the features of this disorder include hypotonia, strabismus, encephalopathy, and cerebellar hypoplasia. According to the Encyclopedia of DNA Elements (ENCODE), this differentially methylated region overlaps a histone 3 lysine 27 acetylation (H3K27Ac) signature of active enhancers in H1 human embryonic stem cells (hESCs), although the effect of altered DNA methylation on this regulatory activity is unclear.

The second region with evidence of parent-of-origin biased methylation in peripheral blood at a Mendelian disease gene overlapped the last intron of the WW-domain-containing oxidoreductase, *WWOX,* on 16q23.1, a gene associated with epileptic encephalopathy (MIM #616211), esophageal squamous cell carcinoma (MIM #133239), and spinocerebellar ataxia (MIM #614322) inherited in the autosomal recessive manner. Interestingly, the methylation pattern in this region appears nearly bimodal, with upd(16)pat samples showing very low levels of methylation in contrast to both upd(16)mat samples and biparental controls, which showed higher levels of methylation. The atypical parent-of-origin biased methylation at this locus might be the result of favored high methylation levels in individuals with at least one maternally methylated template allele, which could serve as a guide for the establishment of methylation on the paternal allele in a hierarchical fashion [44]; however, in the absence of such a template, as is the case in upd(16)pat, the methylation levels might remain low.

Our high-resolution bisulfite sequencing approach has allowed us to closely examine regions of chromosome 16 that are not captured by the methylation arrays commonly used to study differential methylation, thereby allowing us to identify novel regions with potential parent-of-origin biased DNA methylation. Among our candidate regions, we identified loci with evidence of parent-of-origin DNA methylation on 16q24.1 that might contribute to the clinical phenotypes shared between individuals with ACDMPV and upd(16)mat, but also found support for novel differentially methylated parental alleles at other loci. Further studies are needed to validate parent-of-origin biased DNA methylation in the newly identified candidate regions and to evaluate their consequences on the expression of nearby genes. In addition, with expanding sample sizes, we will be able to address differences in methylation that may have resulted from age, gender, or cell type heterogeneity. Alternatively, phenotypic penetrance of some of UPD- or 16q24.1 hemizygosity-linked diseases, suggestive of genomic imprinting, could result also from differential histone 3 methylation and other epigenetic modifications.

## Additional files

Additional file 1: **Table S1.** Overview of available genomic information for upd(16). **Table S2.** Differentially methylated regions identified in blood-based analysis (*n* = 1579), sorted by number of differentially methylated sites. **Table S3.** Differentially methylated regions identified in lung-based analysis (*n* = 8), sorted by number of differentially methylated sites contained. (XLSX 589 kb)

Additional file 2: **Figure S1.** Number of CpG sites in non-overlapping 10 kb windows across chromosome 16 found in hg19 (black) and captured by our bisulfite sequencing method (blue). **Figure S2.** Differential methylation found through blood-based analysis at *NARFL* (S2A), *MGRN1* (S2B), *PMM2* (S2C), *CPPED1* (S2D), *PKD1P1*-centromeric region (S2E), and *WWOX* (S2F). White areas in top panels indicated regions suggestive of parent-of-origin biased methylation based on our analysis, gray areas fall outside these boundaries. Lines in bottom panels were created using LOESS smoothing with a span of 0.1, causing some data points to extend beyond the expected 0–100% methylation range. Abbreviations: B, blood; F, fibroblast; L, lung. **Figure S3.** Differential methylation found through lung-based analysis at *KCNG4* (S3A), *CRISPLD2*/*ZDHHC7*-intergenic region (S3B), *ZDHHC7* (S3C), *KIAA0513* (S3D), intergenic region centromeric to *LINC00311* (S3E), and intergenic region centromeric to *GSE1* (S3F). White areas in indicated regions suggestive of parent-of-origin biased methylation based on our analysis, gray-shaded areas fall outside these boundaries. Top panels show DNA methylation in lung tissue samples; bottom panels show DNA methylation in blood and fibroblast samples for comparison. (DOCX 1442 kb)
