# Supplementary material for: Novel parent-of-origin-specific differentially methylated loci on chromosome 16
Source: Clin Epigenetics. 2019 Apr 8;11:60. doi: 10.1186/s13148-019-0655-8 (PMC6454695; doi:10.1186/s13148-019-0655-8)
Supplement: Supplementary file 2 — Figure S1. Number of CpG sites in non-overlapping 10 kb windows across chromosome 16 found in hg19 (black) and captured by our bisulfite sequencing method (blue). Figure S2. Differential methylation found through blood-based analysis at NARFL (S2A), MGRN1 (S2B), PMM2 (S2C), CPPED1 (S2D), PKD1P1-centromeric region (S2E), and WWOX (S2F). White areas in top panels indicated regions suggestive of parent-of-origin biased methylation based on our analysis, gray areas fall outside these boundaries. Lines in bottom panels were created using LOESS smoothing with a span of 0.1, causing some data points to extend beyond the expected 0–100% methylation range. Abbreviations: B, blood; F, fibroblast; L, lung. Figure S3. Differential methylation found through lung-based analysis at KCNG4 (S3A), CRISPLD2/ZDHHC7-intergenic region (S3B), ZDHHC7 (S3C), KIAA0513 (S3D), intergenic region centromeric to LINC00311 (S3E), and intergenic region centromeric to GSE1 (S3F). White areas in indicated regions suggestive of parent-of-origin biased methylation based on our analysis, gray-shaded areas fall outside these boundaries. Top panels show DNA methylation in lung tissue samples; bottom panels show DNA methylation in blood and fibroblast samples for comparison. (DOCX 1442 kb) [file 13148_2019_655_MOESM2_ESM.docx]

**SUPPLEMENTARY METHODS**

**Patient Descriptions**

*upd(16)mat – blood sample ‘AC’*

AC is an 11 year old male who was referred to genetics for evaluation of congenital heart defect, other congenital anomalies, hemihypertrophy, and some developmental delay. He has complete AV canal defect status post repair and bicuspid aortic valve with dilated aortic root (Z score= +3.3) and ascending aorta (Z-score= +4.8). He developed pectus excavatum after AV canal repair. His abdominal ultrasounds repeatedly showed absent gallbladder.  At 5 years of age, he was found to have a right femur that was longer than the left, with a leg length discrepancy of about 1cm. He initiated screening for Wilms tumor and hepatoblastoma with abdominal ultrasounds every three months and results were unremarkable.

The patient exhibits some learning difficulties. He attends regular classes except for reading and writing where he needs special education classes. He has difficulty focusing and occasionally exhibits arm tapping repetitive movements. He speaks in full sentences but exhibits stuttering and pronunciation difficulties, for which he receives speech therapy.

AC was born at 37 weeks of gestation to a 26-year-old mother via C-section for fetal heart decelerations. His birth weight was 1.8 kg and birth length was 45 cm (both below 1^st^ percentile). He was noted to have a small placenta. Had low pulse ox at 4 days of life, echocardiogram performed which showed AV canal.

Results of exome sequencing revealed maternal uniparental disomy of chromosome 16 including cytogenetic bands 16p13.3p13.13 (hg19 coordinates: 115072-10840847) and bands 16q22.3q24.3 (hg19 coordinates: 73421051-90115921). Of note, no pathogenic variants in genes on chromosome 16 were identified by exome sequencing. In addition, no ACMG secondary findings were identified.

On physical exam at 11 year 5 month old, AC’s weight was at the 63 %ile (Z= +0.32), height at the 86 %ile (Z=+1.08), and his BM at the 43 %ile (Z= -0.18) and OFC at the 40 %ile (Z=-0.17). He had abnormally placed teeth and overcrowded dentition in lower jaw, bifid uvula, thin lips with smooth philtrum.  Chest showed pectus excavatum, with well healed median sternotomy scar. He exhibited hyperlaxity in his fingers and positive thumb sign. Leg length discrepancy of about ~2-3 cm with the right leg longer and lower right thigh circumference was 38 cm (left was 35.5 cm) and upper right calf circumference was 27.5 cm (left was 27 cm). He has a hip tilt when standing and a compensatory curve of his spine but no actual scoliosis. The remainder of physical exam was unremarkable.

*upd(16)mat – blood sample ‘2-0300’*

Female infant born at 36 weeks gestation to a 39yo G6P2 mother with pregnancy complicated by gestational diabetes via vaginal delivery.  Apgars 8, 9 at one and five minutes of age.  Within a few hours of birth, she developed increasing respiratory distress (tachypnea, increased work of breathing) and was placed on CPAP with supplemental oxygen.  Chest radiograph was consistent with hyaline membrane disease.  She received surfactant and was able to be weaned to room air.  However, a few days later, her oxygen requirements increased and chest radiograph showed increased granularity.  She received a second dose of surfactant and was placed back on CPAP.  She continued to require oxygen while on CPAP to maintain saturations. Echocardiogram showed tricuspid regurgitation and pulmonary hypertension for which she was placed on nitric oxide.  Due to the persistent lung disease and increased oxygen requirements, her DNA sample was sent for sequencing for mutations in surfactant-related genes.  She was found to be homozygous for the p.E690K mutation in ABCA3.  Her oxygen requirement increased gradually to 90% FiO2 and she required sedation.  At 2 months of age, she had an acute deterioration in her respiratory condition requiring intubation.  She was transferred to a referral center for infant lung transplantation evaluation.  She remained intubated and mechanically ventilated until bilateral lung transplant at 5 months of age.  Family history negative for surfactant deficiency. Information on ‘2-0300’ was previously published by Wambach et al. (33).

*upd(16)pat – blood samples ‘2-0204’ and ‘2-0225’*

Both previously described by Hamvas et al. (12) as “Patient 2 with ABCA3 deficiency” and “Patient 3 with ABCA3 deficiency”.

*upd(16)mat – fibroblast sample ‘BAB-8048’*

The patient was born prematurely at 32 weeks to a 29 years old G3P2002 Hispanic mother. The pregnancy was complicated by dichorionic-diamniotic twin gestation with significant discordant of fetal growth. There was initially a concern for skeletal dysplasia given shortened limb length and small chest cavity on the proband. However confirmatory genetic testing for skeletal dysplasia was not performed and mother was lost to follow up. The patient was born via emergency C-section due to non-reassuring fetal heart tone. Her APGAR scores were 8 and 9 at 1 and 5 minutes, respectively. Her birth weight, length and head circumference were 578 grams (-3.8 SD), 31 cm (-4.2 SD) and 24.5 cm (-3.2 SD), respectively, while her twin brother’s birth weight was 1979 grams (median). Newborn examination revealed small infant with no obvious dysmorphic findings suggestive of skeletal dysplasia.

Her immediate neonatal course was complicated by respiratory distress, neonatal hypoglycemia and conjugated hyperbilirubinemia concerning for sepsis. However extensive infectious disease work up was negative. She developed non-ketotic hypoglycemia when she was transitioning to enteral feeds. Given persistent prolonged hypoglycemia despite high glucose infusion rate, she had extensive endocrinology and metabolic evaluation. During one of the hypoglycemic episodes, she was found to have markedly elevated growth hormone (GH) with normal insulin level and low normal cortisol level raising concern for possible GH resistance or cortisol deficiency. However, she had normal brain MRI ruling out septo-optic dysplasia. Her ACTH stimulation test, newborn screening and acylcarnitine profile were normal. Her hypoglycemia resolved with time without recurrences and no further diagnostic testing was performed.

She developed conjugated hyperbilirubinemia in the first two weeks of life with a direct bilirubin level of 14.4 mg/dl at day of life 10. She also had persistently elevated ALT, AST and GGT with normal synthetic liver function. Differential diagnosis was quite broad included transient cholestasis of newborn, TPN induced cholestasis, inborn error of metabolism (particularly tyrosinemia and galactosemia), alpha 1 antitrypsin deficiency, biliary atresia, Allagile syndrome and familial intrahepatic cholestasis. Extensive work up to reveal potential causes of neonatal cholestasis were negative. Abdominal ultrasound showed normal liver and gallbladder. HIDA scan showed normal bile acids flow ruling out biliary atresia. Urine organic acid analysis revealed no excretion of succinylacetone. She also had negative mutation on jaundice chip (*SERPINA1, JAG1, ATP8B1, ABCB11 and ABCB4*). She was started on conservative management with ursodiol and vitamin ADEK. Her direct bilirubin and GGT trended down throughout her hospital course and normalized at 3 months old but she continued to have mildly elevated ALT, AST and alkaline phosphatase. Repeat liver ultrasound at 7 months old showed hepatomegaly.

She had severe feeding difficulties with poor oral motor skills and required enteral feeding. She was also treated for gastro-esophageal reflux disease and had a fundoplication performed at 4 months old. She showed good catch up growth for her weight while on high calorie formula however her linear growth was lagging. At 9 months old, her weight was 7.14 kg (-1 SD), length was 59.1 cm (> -4 SD) and head circumference was 41.3 cm (-2 SD). Global developmental delay especially in gross motor skills became apparent with time although improving. At 9 months old she had good head control but unable to roll over and sit without support. She received intensive physical and occupational therapy. She had minor dysmorphic features with bilateral epicanthal folds and bulbous nasal tip.

*upd(16)mat – fibroblast sample ‘BAB-8047’ (excluded from analysis after data QC)*

The patient was born at 36 weeks gestation to a 40 years old G2P1001 Caucasian mother via repeat C-section. Parents are non-consanguineous. Prenatal history was remarkable only for IUGR noted at the beginning of second trimester. Her birth weight and length were 1,360 grams (-2.8 SD) and 40.6 cm (-2.8 SD), respectively. Neonatal history was complicated by neonatal jaundice with onset at one week of life. Due to suspected biliary atresia, she had extensive GI evaluation including liver biopsy, HIDA scan and cholangiography which were all normal. Jaundice reportedly resolved at approximately two months of life with conservative management. No further diagnostic work up was attempted.

In the first one year of life, she developed recurrent lower respiratory tract infection prompting an airway evaluation. Airway evaluation with CT chest and bronchoscopy revealed that she had left lung hypoplasia and recurrent pulmonary hemorrhages due to ectatic alveolar blood vessels. Additionally, echocardiography revealed mild pulmonary hypertension, likely because of diffusely small pulmonary artery.

She also developed feeding difficulties and poor weight gain throughout her life although had normal growth velocity. She initially required enteral feeds but was able to transition to oral feeding with intensive speech therapy. At six years old, both weight and height were still below third percentiles.

*ACDMPV – lung sample ‘60.4’*

Previously described by Szafranski et al. (32).

*ACDMPV – lung sample ‘115.3’*

Previously described by Szafranski et al. (29).

**SUPPLEMENTARY FIGURES**


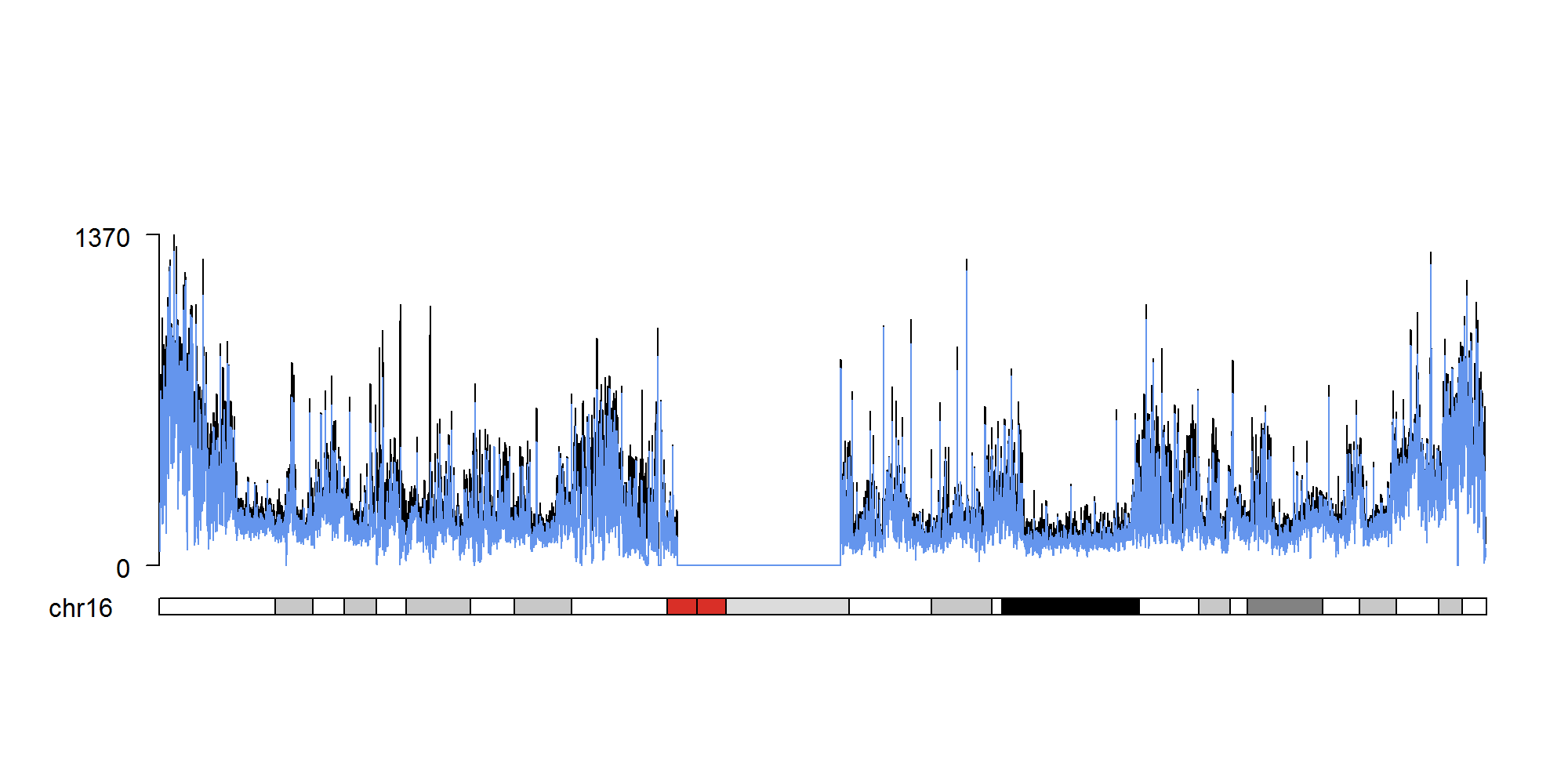


**Supplementary Figure S1.** Number of CpG sites in non-overlapping 10 kb windows across chromosome 16 found in hg19 (black) and captured by our bisulfite sequencing method (blue).


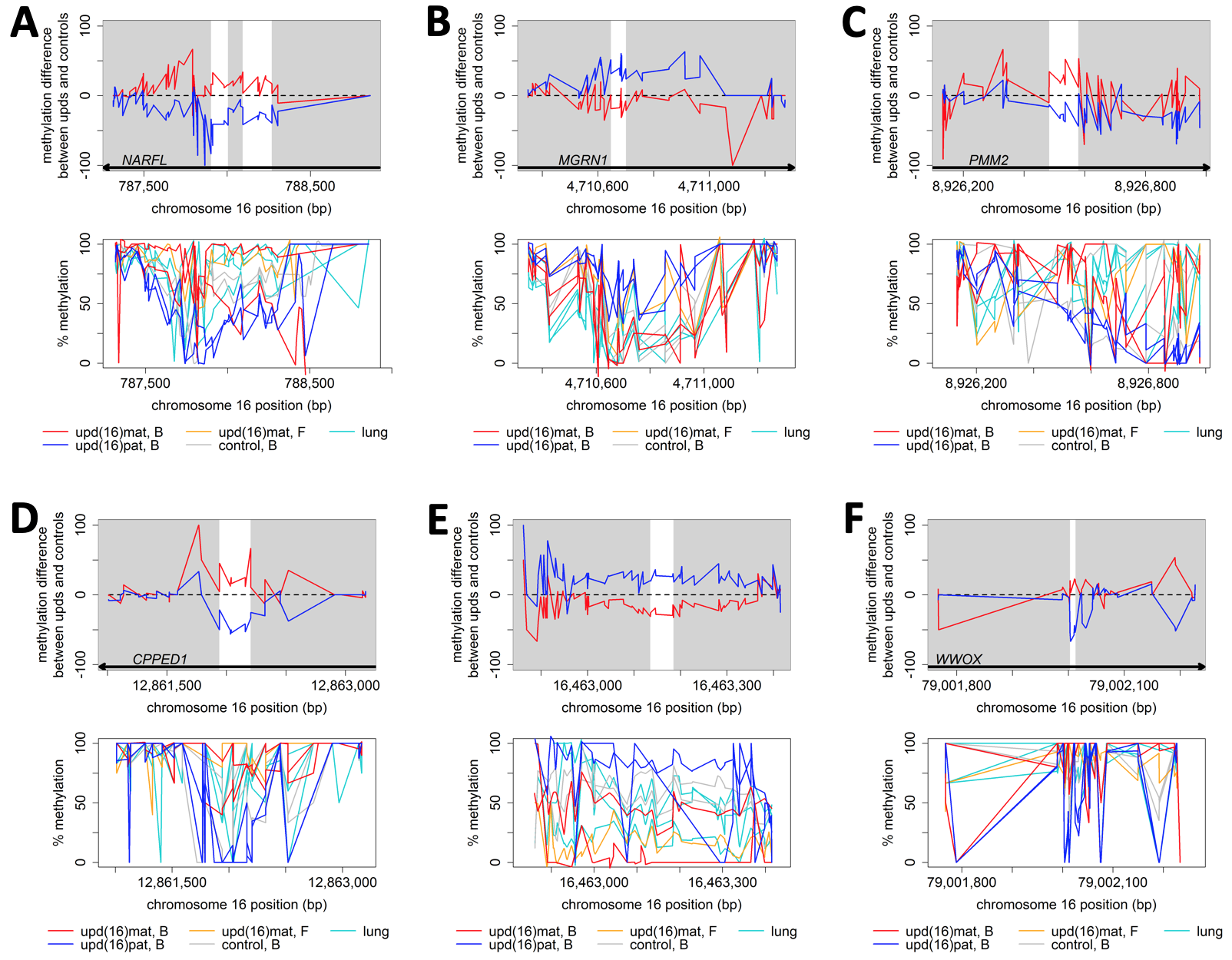


**Supplementary Figure S2.** Differential methylation found through blood-based analysis at *NARFL* (**S2A**), *MGRN1* (**S2B**), *PMM2* (**S2C**), *CPPED1* (**S2D**), *PKD1P1*-centromeric region (**S2E**), and *WWOX* (**S2F**). White areas in top panels indicated regions suggestive of parent-of-origin biased methylation based on our analysis, grey areas fall outside these boundaries. Lines in bottom panels were created using LOESS smoothing with a span of 0.1, causing some data points to extend beyond the expected 0-100% methylation range. Abbreviations: B, blood; F, fibroblast; L, lung.


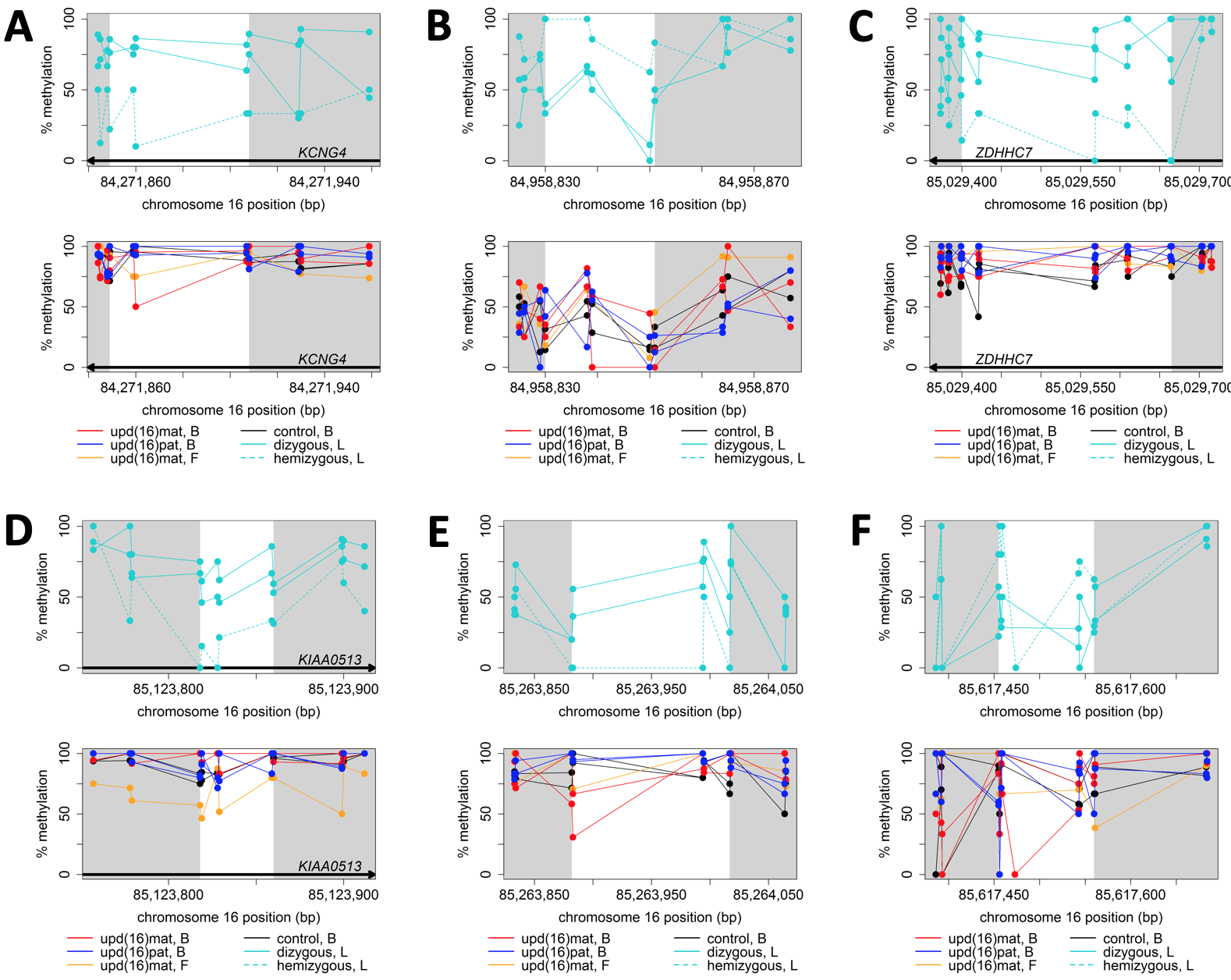


**Supplementary Figure S3**. Differential methylation found through lung-based analysis at *KCNG4* (**S3A**), *CRISPLD2*/*ZDHHC7*-intergenic region (**S3B**), *ZDHHC7* (**S3C**), *KIAA0513* (**S3D**), intergenic region centromeric to *LINC00311* (**S3E**), and intergenic region centromeric to *GSE1* (**S3F**). White areas in indicated regions suggestive of parent-of-origin biased methylation based on our analysis, grey-shaded areas fall outside these boundaries. Top panels show DNA methylation in lung tissue samples; bottom panels show DNA methylation in blood and fibroblast samples for comparison.
